# Developmental differences in effects of task pacing on implicit sequence learning

**DOI:** 10.3389/fpsyg.2014.00153

**Published:** 2014-02-25

**Authors:** Amanda S. Hodel, Julie C. Markant, Sara E. Van Den Heuvel, Jenie M. Cirilli-Raether, Kathleen M. Thomas

**Affiliations:** ^1^Institute of Child Development, University of MinnesotaMinneapolis, MN, USA; ^2^Department of Cognitive, Linguistic, and Psychological Sciences, Brown UniversityProvidence, RI, USA

**Keywords:** implicit sequence learning, serial reaction time paradigm, statistical learning, probabilistic learning, developmental invariance hypothesis

## Abstract

Although there is now substantial evidence that developmental change occurs in implicit learning abilities over the lifespan, disparate results exist regarding the specific developmental trajectory of implicit learning skills. One possible reason for discrepancies across implicit learning studies may be that younger children show an increased sensitivity to variations in implicit learning task procedures and demands relative to adults. Studies using serial-reaction time (SRT) tasks have suggested that in adults, measurements of implicit learning are robust across variations in task procedures. Most classic SRT tasks have used response-contingent pacing in which the participant's own reaction time determines the duration of each trial. However, recent paradigms with adults and children have used fixed trial pacing, which leads to alterations in both response and attention demands, accuracy feedback, perceived agency, and task motivation for participants. In the current study, we compared learning on fixed-paced and self-paced versions of a spatial sequence learning paradigm in 4-year-old children and adults. Results indicated that preschool-aged children showed reduced evidence of implicit sequence learning in comparison to adults, regardless of the SRT paradigm used. In addition, we found the preschoolers showed significantly greater learning when stimulus presentation was self-paced. These data provide evidence for developmental differences in implicit sequence learning that are dependent on specific task demands such as stimulus pacing, which may be related to developmental changes in the impact of broader constructs such as attention and task motivation on implicit learning.

## Introduction

Implicit learning has been defined broadly as a sensitivity to patterns, regularities, sequential information, and/or statistical dependencies in the environment that largely takes place outside of conscious awareness (see reviews by Cleeremans et al., 1998; Perruchet and Pacton, 2006). Early theories of implicit learning postulated that because implicit learning is a fundamental, adaptive cognitive mechanism, it should recruit evolutionarily basic brain regions and demonstrate early maturation (Reber, 1993). Subsequent research has confirmed that implicit learning mechanisms do generally utilize so-called evolutionarily primitive brain regions such as the basal ganglia (e.g., Rauch et al., 1997; Bischoff-Grethe et al., 2004) and do appear early in development (i.e., during infancy, see Canfield and Haith, 1991; Saffran et al., 1997; Kirkham et al., 2002). Additionally, there is a growing body of evidence that developmental change occurs in implicit learning abilities over the lifespan (e.g., Mayberry et al., 1995; Fletcher et al., 2000; Clohessy et al., 2001; Thomas and Nelson, 2001; Thomas et al., 2004; Vaidya et al., 2007; Janacsek et al., 2012; however, see also Meulemans et al., 1998; Vinter and Perruchet, 2000, 2002; Dorfberger et al., 2007; Karatekin et al., 2007; Amso and Davidow, 2012 for developmental invariance of implicit learning arguments). However, describing the specific developmental trajectory of implicit learning skills during childhood remains challenging given the wide variety of tasks and age groups utilized across studies.

In the adult implicit learning literature, the classic serial-reaction time (SRT) task (Nissen and Bullemer, 1987) remains one of the most widely accepted measures of implicit learning. In the classic SRT task participants are required to map the spatial location of a visual target to a spatially corresponding motor response (e.g., button press). During the task, the appearance of target stimuli is alternately constrained to follow a repeating pattern or a set of probabilistic rules, or determined randomly. Participants are typically unable to verbally describe the repeating pattern that is present. However, over time individuals show evidence of sequence specific learning, as demonstrated by faster reaction times on sequence trials in comparison to random trials.

Results from SRT tasks with adults have suggested that measurements of this form of implicit learning are robust across many variations in task procedures. Importantly, sequence specific learning is observed under different sequence probability structures (e.g., Stadler, 1992), as well with variations in the cue dimensions conveying the sequence (e.g., spatial location of sequence vs. stimulus identity; Robertson and Pascual-Leone, 2001). Learning is also observed in paradigms where the structure of the random trials is modified, including in paradigms when random trials are never present and the sequence is continuously cycled (Nissen and Bullemer, 1987), when random trials are introduced between sequence repetitions (Meulemans et al., 1998; Chambaron et al., 2006), and when random trials appear probabilistically within the repeated sequence (e.g., Song et al., 2007). Furthermore, sequence specific learning is also observed when motor response demands of the traditional SRT task are altered to break simple stimulus-response mappings (Chambaron et al., 2006; Deroost and Soetens, 2006).

Although SRT tasks are a widely accepted measure of implicit learning in adults, only a small number of studies have been conducted investigating implicit learning using this paradigm in children or adolescents. In the first developmental SRT study published, Meulemans et al. (1998) did not find age-related differences in implicit sequence learning among 6- and 10-year-old children and adults. This result was extended into older age ranges by Karatekin et al. (2007) who failed to find significant age related differences among children, adolescents, and young adults in manual or oculomotor measures of implicit learning.

However, other researchers have found strikingly different results while using highly similar SRT paradigms with children. For example, Thomas and Nelson (2001) found that while 4-, 7-, and 10-year-old children did not differ by age group in magnitude of implicit sequence learning, the number of individuals demonstrating evidence of sequence specific learning increased with age. Furthermore, a later fMRI study found that in comparison to adults, children age 7–11 years demonstrated reduced magnitude of overall sequence learning, required more time on task to show a significant learning effect, and showed greater recruitment of subcortical motor circuitry during SRT task performance, all suggesting general developmental changes in both neural and behavioral components of implicit learning from childhood to adulthood (Thomas et al., 2004). Additional research with SRT paradigms using both unimanual and bimanual task versions (De Guise and Lassonde, 2001), assessing the role of sleep in sequence learning consolidation (Fischer et al., 2007), and tracking SRT performance over multi-day training (Savion-Lemieux et al., 2009) have provided at least some indication that implicit learning abilities as measured by the SRT task are not developmentally invariant.

One possible reason for the discrepant results across SRT studies may be that younger children show an increased sensitivity to variations in implicit learning task procedures and demands relative to adults. In particular, one procedural variation in SRT tasks that has remained largely uninvestigated in both adults and children that may have a strong impact on children's learning is task pacing. SRT tasks traditionally have used response-contingent pacing in which the participant's own reaction time determines the duration of each individual trial. However, to control for total stimulus exposure and overall task duration across participants, many researchers have also begun to use fixed-trial pacing, particularly in fMRI studies utilizing SRT tasks (e.g., Rauch et al., 1997; Bischoff-Grethe et al., 2004; Thomas et al., 2004). The introduction of fixed-paced trials may lead to unintentional alterations in both response and attention demands, accuracy feedback, perceived agency, and task motivation for participants. To our knowledge no study has specifically investigated the role of task pacing (i.e., self- vs. fixed-paced trial durations) on developmental differences in sequence learning as measured via the SRT paradigm.

The purposes of the present study were: (1) to replicate previous findings of developmental differences in implicit learning as measured by the SRT task and (2) to investigate whether variations in task pacing impact implicit learning on the SRT task equivalently in children and adults. Given that the greatest developmental differences in implicit learning are likely to exist between the youngest testable age group of children vs. adults, we directly compared learning of 4-year-old children and adults. In experiment 1, group differences in learning were assessed on self- and fixed-paced versions of a spatial sequence learning SRT paradigm. In experiment 2, we separately assessed whether response contingent feedback (based on accuracy) influenced children's learning in the context of the self-paced task. Overall, we hypothesized that 4-year-old children would show reduced learning relative to adult participants regardless of the SRT paradigm used. In addition, we hypothesized that preschoolers would show increased sensitivity to SRT task demands, such that children would exhibit reduced learning when the rate of stimulus presentation was fixed and not contingent on their own response time.

## Experiment 1

### Materials and methods

#### Participants

Data from 60 preschoolers (*M*_age_ = 4.74 years; range = 4.10–5.00 years; 30 female) and 60 adults (*M*_age_ = 23.14 years; range = 18.28–34.27 years; 30 female) were analyzed in the final sample. Families of children were recruited from a community volunteer participant pool maintained by the University of Minnesota. Children in the final sample were predominantly Caucasian (95%), lived in college-educated (93%) two-parent families (98%), with median incomes between $76,000 and $100,000. Adult participants were recruited from the University of Minnesota campus. Adults in the final sample were predominantly Caucasian (80%) and the majority were undergraduate or post-graduate students (78%). All participants were prescreened to exclude any history of birth complications (including premature birth), serious medical issues, learning disabilities, or personal or immediate family history of neurological and/or psychological disorders. Parents of children provided consent to participate and were compensated for their efforts. Children received a small gift (e.g., book, set of stickers, stuffed animal, puzzle) selected from a cabinet of prizes after completion of the experiment. Adult participants also provided consent and were compensated for study participation. Study procedures were approved by the University of Minnesota's Institutional Review Board.

Additional participants were tested but not included in the final sample due to poor task accuracy (34 preschoolers), explicit awareness of the sequence (3 preschoolers, 1 adult), and failure to complete the task (12 preschoolers, 1 adult; see Procedure for more detail).

#### Procedure

**Figure 1:**
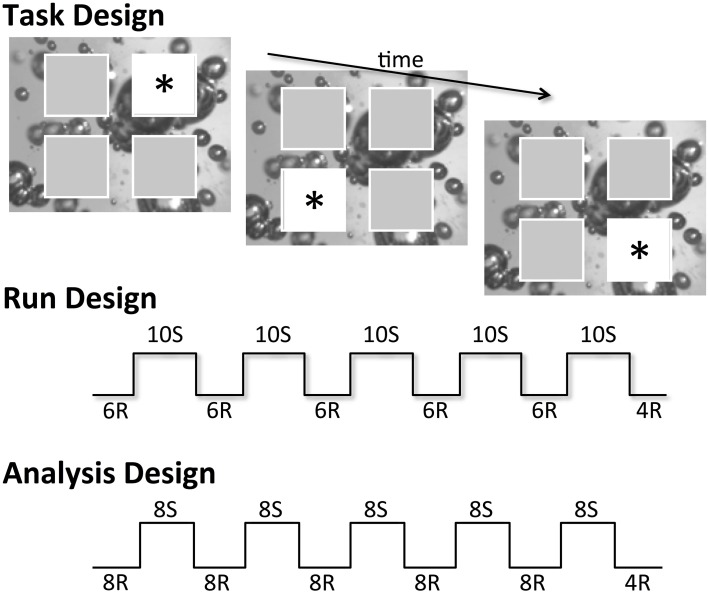
**Basic task, run, and analysis design used for each of the five runs of the SRT task variants, where R represents pseudorandomly appearing stimuli and S denotes stimuli that followed the 10-item spatial sequence.** Stimuli are denoted by an asterisk; actual stimuli used were cartoon characters from the children's television program Sesame Street.

Child and adult participants were told they would be playing a computerized game of tag with the characters from Sesame Street to investigate how children learn new skills with practice. During each experimental trial, a 9 × 9 cm image of the face of one of four Sesame Street characters was presented in a framed location on a 41 × 31 cm monitor. The four frames were arranged in a quadrant orientation, with a separate 9 × 9 cm frame in each quadrant of the screen; frames were spaced 7 cm apart horizontally and 6 cm apart vertically (see Figure 1). Participants were instructed to “tag” the character as quickly as possible by pressing a button that corresponded to the character's spatial location on a button response box, while making as few mistakes as possible. Participants were instructed to use their dominant hand to respond (4-year-olds completed a brief test with the experimenter prior to beginning the experiment to determine handedness). The 35.5 × 35.5 cm button box consisted of four large 5 × 5 cm buttons that corresponded to the arrangement of the spatial locations on the computer screen. Between trials, participants were encouraged to return their hand to a neutral, central position on the button box. Button presses were collected and evaluated for accuracy of first press and reaction time on correct trials.

All participants completed five runs of 84 trials with the potential for short breaks between runs. Prior to beginning the experimental blocks, children also completed one or two short practice runs to ensure task comprehension. Each practice block was composed of 36 pseudorandomly ordered trials constrained such that stimuli never appeared consecutively within the same frame and were balanced to include an equal probability of target appearance in all frame locations.

Participants were randomly assigned to complete either a fixed-paced or a self-paced version of the SRT task. In the fixed-paced condition, each stimulus was presented on the screen for a constant trial duration, followed by a fixed inter-stimulus interval (ISI), without feedback regarding participant accuracy. Trial duration for adults was 750 ms with an ISI of 750 ms based on previous studies in our laboratory with adults and 8-year-old children. However, initial testing with 4-year-olds suggested that the trials were too rapid for many of the children. Based on the average response time for these pilot participants, we increased the stimulus duration to 1500 ms for 4-year-olds, with an ISI of 1500 ms. In the self-paced condition, each stimulus was instead presented for a variable trial duration, followed by a fixed response-to-stimulus interval (RSI; 500 ms for both adults and children), with the trial duration contingent upon the time taken for the participant to execute a correct button press response.

Within each of the five runs, participants were presented with interleaved blocks of pseudorandom and sequence trials (see Figure 1). Sequence blocks were composed of 10 trials following a fixed, 10-step sequence of spatial locations. Each participant was exposed to only one of two possible predictable sequences during the task, with the two sequence variants counterbalanced across task condition, age group, and gender. The first sequence followed the pattern 3-1-4-2-1-3-4-1-2-4, where 1 represents the top left frame, 2 represents the top right frame, 3 represents the bottom left frame, and 4 represents the bottom right frame. The alternate sequence followed the pattern 1-3-2-4-3-1-2-3-4-2. Pseudorandom blocks consisted of six trials without any fixed pattern of spatial locations. Pseudorandom blocks were constrained such that stimuli never appeared consecutively within the same frame and were balanced to match the uneven location probabilities presented in the sequence blocks. No salient cue marked the switch from pseudorandom to repeated sequence blocks, and participants were not informed that the stimuli sometimes followed a hidden sequence.

Directly following completion of the entire task, children and adults were asked a series of open-ended, free-recall questions to assess potential explicit awareness of the sequence structure, including: What did you think of the game? Did you feel like you got faster with practice? Did you ever think you could tell where the pictures were going to move next? If children or adults indicated the presence of a pattern structure by responding “yes” or “maybe” to the final question, they were encouraged to generate any patterns by demonstrating them on the original button box or an example figure that depicted the four frame locations on the computer screen.

Additional participants were tested but excluded from the final sample of 60 preschoolers and 60 adults. Data from 1 adult (1 fixed-paced) and 12 preschoolers (5-self paced, 7 fixed-paced) were excluded due to failure to complete five runs of the task. In order to effectively compare learning across children and adults and to ensure learning was implicit, we made an effort to include participants in the final sample who reached similar levels of behavioral performance and explicit awareness of the sequence. All adults achieved an average accuracy above 80 percent on random and sequence trials across the entire task. Preschoolers were included in the final sample if they attained the same level of accuracy, or if their average accuracy within each individual run on random and sequence trials separately was at least 70 percent. Thirty-four preschoolers failed to meet this accuracy criterion (12 self-paced, 22 fixed-paced). Explicit awareness of the sequence was coded as recall of a consecutive string of five or more sequential locations or recall of two strings of four or more consecutive locations, even if overlapping (Thomas et al., 2004). Data from one adult (1 self-paced) and three preschoolers (1 self-paced, 2 fixed-paced) were removed due to explicit sequence awareness. Exploratory analyses using a more sensitive measure of partial sequence awareness, coded as recall of a consecutive string of three or four sequential locations, indicated that children were more likely to exhibit partial awareness than adults, χ^2^_(1, 120)_ = 5.78, *p* < 0.02. However, partial awareness of the sequence was not associated with improvements in implicit learning, and did not account for differences by task or age group; thus these individuals were retained in the final data set given that they did not differentially impact the reported results.

#### Data preprocessing

Due to the partially ambiguous structure of the 10-item sequences used, two preceding locations in serial order were required to accurately predict the third sequence item. Thus, because the first two trials of the 10-item sequence were unpredictable, these trials were coded as random (following Meulemans et al., 1998), resulting in an analysis plan comparing blocks of eight random trials or eight sequence trials. Furthermore, the four random trials at the end of each run were also excluded from analyses; these trials were present in the task design only to ensure runs did not end following a block of sequence trials, which could inadvertently increase the salience of the hidden sequence to participants (see Figure 1).

Additionally, since response time was unconstrained in the self-paced condition, data from the 4-year-old children were filtered based on a cut-off of 2.5 standard deviations above the individual's mean reaction time on correct trials. This filtering removed a small number of trials with atypically long latencies that resulted from off-task behavior. This filter was not used for the data of preschoolers in the fixed-paced condition because children's maximum reaction time on this task was restricted by the fixed-length trial duration.

#### Statistical analyses and learning measure

Planned analyses first investigated potential effects of age or task condition on accuracy for random and sequence trials using 2 × 2 × 2 repeated measures ANOVAs with trial type (random, sequence), age group (preschooler, adult), and task (fixed-paced, self-paced) as independent variables.

Because we anticipated large age-related differences in both average reaction time and reaction time variability, we used a z-normalized reaction time score based on each individual's mean (following Thomas et al., 2004). Using this approach, learning was measured by the mean difference between standardized reaction times on random trials in comparison to sequence trials. Successful sequence learning was thus indicated by a positive mean difference score, averaged across runs (i.e., overall learning effect), which reflected faster reaction times on sequence trials in comparison to random trials. Data based on raw reaction time scores are also presented.

Developmental differences in implicit learning by task condition were assessed using a 2 × 2 ANOVA with age group (preschooler, adult) and task (fixed-paced, self-paced) as independent variables and z-normalized learning scores as the dependent variable. Simple task-related effects within groups were also computed using independent samples *t*-tests. Finally, one-sample *t*-tests were then used to verify whether participants demonstrated statistically significant learning (i.e., a z-normalized learning score greater than 0) within specific age group and task conditions. All analyses were conducted at α = 0.05. Results are plotted using means and standard errors.

### Results

#### Accuracy

Adults and preschoolers in the final sample demonstrated high levels of behavioral accuracy on both random (adults: *M* = 0.99, *SD* = 0.01; preschoolers: *M* = 0.89, *SD* = 0.05) and sequence trial types (adults: *M* = 0.99, *SD* = 0.00; preschoolers: *M* = 0.90, *SD* = 0.05). A 2 × 2 × 2 repeated measures ANOVA with trial type, age group, and task as independent variables and accuracy as the dependent variable revealed a significant main effect of trial type, *F*_(1, 116)_ = 4.42, *p* = 0.04, η_partial^2^_ = 0.04, where performance was better overall on sequence than random trials. Unsurprisingly, there was also a significant main effect of age such that adults performed more accurately than children, *F*_(1, 116)_ = 271.66, *p* < 0.01, η_partial^2^_ = 0.70. As previously described, children who had poor response accuracy overall were excluded from analyses. Therefore, the age effect observed here is likely a more conservative estimate than would be expected in a more inclusive sample of 4-year-old children.

Importantly, there was no main effect of task, *F*_(1, 116)_ = 0.14, *p* = 0.71, η_partial^2^_ = 0.00, no significant interaction between age and task, *F*_(1, 116)_ = 0.30, *p* = 0.58, η_partial^2^_ = 0.00, and no other significant two or three way interactions, indicating that although adults were more accurate than children, this was a global accuracy benefit that occurred equivalently across trial types and task conditions.

#### Learning

Preliminary analyses using a 2 × 2 repeated measures ANOVA with trial type and age group as independent variables and reaction time as the dependent variable revealed a significant main effect of age, *F*_(1, 118)_ = 405.12, *p* < 0.01, η_partial^2^_ = 0.77. Adults responded more quickly than children across trial types, justifying our use of the z-normalized learning measure to compare learning between age groups that differed substantially in reaction time and reaction time variability (see Statistical Analyses and Learning Measure for more detail). Below we reported results for z-normalized reaction times but summarized results using raw reaction times are also included for comparison.

**Figure 2:**
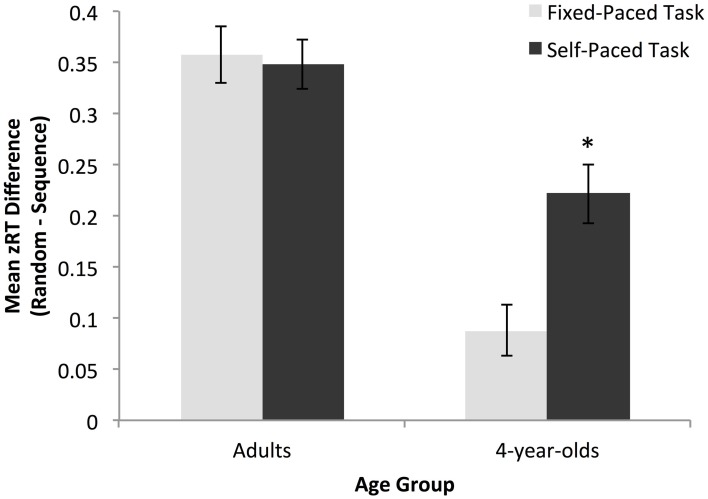
**Magnitude of the overall learning effect for adults and preschoolers in the fixed- and self-paced conditions.** Adults showed equivalent learning across tasks while preschoolers had significantly greater learning scores in the self-paced condition. ^*^p < 0.05.

Developmental differences in implicit learning by task condition were assessed using a 2 × 2 ANOVA with age group and task as independent variables and z-normalized learning score as the dependent variable. There was a significant main effect of group, *F*_(1, 116)_ = 55.90, *p* < 0.01, η_partial^2^_ = 0.33, where adults showed higher levels of sequence specific learning than children across task conditions (adults: *M* = 0.35, *SD* = 0.14; preschoolers: *M* = 0.15, *SD* = 0.16). There was also a significant main effect of task, *F*_(1, 116)_ = 5.49, *p* = 0.02, η_partial^2^_ = 0.05, where learning was greater in the self-paced than the fixed-paced condition (self-paced: *M* = 0.28, *SD* = 0.16; fixed-paced: *M* = 0.22, *SD* = 0.20). Finally, as predicted, we found a significant interaction between age group and task, indicating that the effect of task condition on learning was greater for children than adults, suggesting that younger children showed an increased sensitivity to variations in the SRT task procedure and demands, *F*_(1, 116)_ = 7.34, *p* = 0.01, η_partial^2^_ = 0.06 (see Figure 2).

Importantly, follow-up analyses utilizing one-sample *t*-tests on the z-normalized learning measure indicated that both adults and 4-year-olds showed a robust, overall learning effect on both tasks versions [adult fixed-paced: *M* = 0.36, *SD* = 0.15, *t*_(29)_ = 12.82, *p* < 0.01; adult self-paced: *M* = 0.35, *SD* = 0.13, *t*_(29)_ = 14.42, *p* < 0.01; preschooler fixed-paced: *M* = 0.09, *SD* = 0.13, *t*_(29)_ = 3.58, *p* < 0.01; preschooler self-paced: *M* = 0.22, *SD* = 0.16, *t*_(29)_ = 7.60, *p* < 0.01]. However, while adults showed no difference in overall learning magnitude between the fixed- and self-paced tasks, *t*_(58)_ = 0.26, *p* = 0.79, children's learning was significantly greater in the self-paced condition, *t*_(58)_ = −3.52, *p* < 0.01.

When the identical analyses were conducted using raw reaction time learning scores highly similar effects were observed. The main effect of age group was now non-significant because of large overall differences between adults and 4-year-olds in baseline reaction time, *F*_(1, 116)_ = 2.92, *p* = 0.09, η_partial^2^_ = 0.03. However, the main effect of task remained, *F*_(1, 116)_ = 12.85, *p* < 0.01, η_partial^2^_ = 0.10, with greater learning in the self-paced than the fixed-paced condition (self-paced: *M* = 72.30 ms, *SD* = 57.02 ms; fixed-paced: *M* = 41.03 ms, *SD* = 40.07 ms). Similarly, follow-up analyses using one-sample *t*-tests on the raw reaction time learning scores indicated that both age groups showed significant learning on all task versions (adult fixed-paced: *M* = 44.91 ms, *SD* = 21.34 ms, *t*_(29)_ = 11.53, *p* < 0.01; adult self-paced: *M* = 53.53, *SD* = 24.36 ms, *t*_(29)_ = 12.04, *p* < 0.01; preschooler fixed-paced: *M* = 37.16 ms, *SD* = 52.73 ms, *t*_(29)_ = 3.86, *p* < 0.01; preschooler self-paced: *M* = 91.06 ms, *SD* = 72.75 ms, *t*_(29)_ = 6.85, *p* < 0.01). Most importantly, the interaction between age group and task remained significant, *F*_(1, 116)_ = 6.74, *p* = 0.01, η_partial^2^_ = 0.06, such that children, *t*_(58)_ = 3.29, *p* < 0.01, but not adults, *t*_(58)_ = 1.46, *p* = 0.15, showed a difference in learning magnitude by task condition.

The time courses of z-normalized and raw reaction times for random and sequence trials by age group and task are presented in the supplementary materials (see supplementary figures 1–4).

### Interim discussion

Results from adult participants support the conclusions of previous research suggesting that implicit learning measures are robust across variations in SRT task procedures. Specifically, adults showed equivalent learning on both self- and fixed-paced versions of a spatial sequence learning SRT paradigm. However, results from 4-year-old children instead indicated that preschoolers showed significantly greater learning on a self-paced SRT task in comparison to a fixed-paced SRT task, suggesting implicit learning measures in younger children are highly sensitive to changes in task demands.

Why did 4-year-old children demonstrate dramatically larger learning effects on the self-paced task? Although the fixed- and self-paced tasks differed in stimulus pacing, these two tasks also differed in whether accuracy feedback was provided to the participant. Specifically, in the fixed-paced task, each stimulus was presented for a constant trial duration without feedback regarding participant accuracy. However, in the self-paced condition, accuracy feedback was intrinsically present in the task design given that each stimulus was presented for a variable trial duration based on the amount of time required by the participant to execute the correct response. Thus, although children showed greater learning in the self-paced condition, it was unclear whether this was due specifically to task pacing, accuracy feedback, or more general attention/motivational factors.

## Experiment 2

The purpose of experiment 2 was to separately assess the impact of accuracy feedback on implicit learning measures in the context of the self-paced SRT task. Given that adults showed equivalent learning across the two task variants utilized in experiment 1, we chose to examine the effect of accuracy feedback on implicit learning only in preschool-aged children. We developed a separate version of the self-paced task that was non-contingent on response accuracy to assess the impact of accuracy feedback on implicit learning in this context. We then compared performance on this non-contingent task variant to performance on the two tasks in Experiment 1.

### Materials and methods

#### Participants

Data from 30 preschoolers (*M*_age_ = 4.69 years; range = 4.03–4.91 years; 15 female) recruited via the same method as in experiment 1 were analyzed as the final sample. Additional children were tested and excluded from the final sample due to failure to reach the accuracy criterion (3 children), explicit awareness of the sequence (2 children), or failure to complete the task (3 children) based on the same criteria set in experiment 1.

#### Procedure

Children completed a modified version of the self-paced task described in experiment 1, which was designed to be non-contingent on response accuracy. In this non-contingent self-paced condition, each stimulus was presented for a variable trial duration, followed by a fixed RSI (500 ms). Trial duration was contingent upon the time taken for the participant to execute any button press, regardless of accuracy.

Explicit awareness was assessed via the same procedure as described in experiment 1. Because reaction time was not constrained in this task, data were filtered in the same manner as described in experiment 1 using a 2.5 standard deviation cut-off and analyzed using identical statistical methods.

### Results

#### Accuracy

On the non-contingent self-paced task, preschoolers demonstrated high levels of behavioral accuracy on both random (*M* = 0.91, *SD* = 0.04) and sequence (*M* = 0.92, *SD* = 0.05) trials, although accuracy was again marginally higher for sequence trials, *t*_(30)_ = −1.96, *p* = 0.06. A 2 × 3 mixed measures ANOVA indicated that there was no significant difference in preschooler's average accuracy over random and sequence trials between the fixed-paced (experiment 1), self-paced (experiment 1), or non-contingent self-paced (experiment 2) tasks, *F*_(2, 87)_ = 2.79, *p* = 0.07, ηpartial^2^ = 0.06.

#### Learning

Z-normalized results indicated that preschoolers demonstrated a robust overall learning effect on the non-contingent self-paced task (*M* = 0.20, *SD* = 0.16, *t*_(29)_ = 6.75, *p* < 0.01).

**Figure 3:**
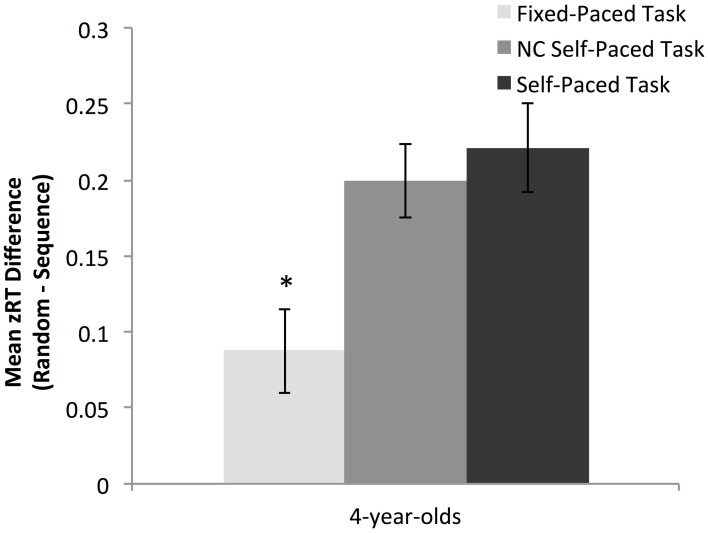
**Magnitude of the overall learning effect for preschoolers across task (data from experiment 1 and 2).** Learning for preschoolers was equivalent on the two self-paced tasks and significantly greater than learning in the fixed-paced condition. ^*^p < 0.05.

To isolate the effects of task pace on learning, preschoolers' learning was compared on the fixed-paced and non-contingent self-paced task, which differed only in rate of stimulus presentation. An independent samples *t*-test indicated that preschoolers showed greater learning on the non-contingent self-paced task in comparison to the fixed-paced task, *t*_(58)_ = 2.91, *p* = 0.01 (see Figure 3).

To examine the effects of accuracy feedback on learning, preschoolers' learning was compared on the self-paced and non-contingent self-paced tasks, which differed only in whether a correct response was required to advance through trials. An independent samples *t*-test indicated that preschoolers showed equivalent overall learning on the two self-paced task versions, *t*_(58)_ = −0.54, *p* = 0.59 (see Figure 3).

Effects using raw reaction time learning scores rather than z-normalized learning scores were equivalent. A one-sample *t*-test indicated that preschoolers showed significant learning on the non-contingent self-paced task (*M* = 78.72 ms, *SD* = 59.57 ms, *t*_(29)_ = 7.24, *p* < 0.01). Learning based on raw reaction time scores on this task was greater in comparison to the fixed-paced task, *t*_(58)_ = 2.86, *p* < 0.01, but equivalent to the original self-paced task version, *t*_(58)_ = −0.72, *p* = 0.48.

The time courses of z-normalized and raw reaction times for random and sequence trials on the non-contingent self-paced task are presented in the supplementary materials (see supplementary figures 5, 6).

## Discussion

### General developmental differences

Overall, results support a growing literature (e.g., Mayberry et al., 1995; Fletcher et al., 2000; Clohessy et al., 2001; Thomas and Nelson, 2001; Thomas et al., 2004; Vaidya et al., 2007; Janacsek et al., 2012) suggesting that developmental change occurs in implicit learning during childhood. Specifically, we found that implicit learning, as measured by the classic SRT task, is not equivalent in preschool-aged children and adults. Instead, across fixed-, self-paced, and non-contingent self-paced task variants, 4-year-old children showed reduced sequence specific learning in comparison to adults. Furthermore, these developmental differences in learning were present despite high levels of task accuracy in both 4-year-olds and adults, suggesting a general immaturity of implicit learning skills in young children.

Previous authors have argued that developmental differences in implicit learning observed in some SRT studies may be due to group differences in explicit awareness of the sequence structure rather than true differences in implicit learning abilities (e.g., Meulemans et al., 1998). In the present study, this problem is unlikely given that any participants who demonstrated explicit awareness of the underlying sequence structure were removed prior to final data analyses. In addition, more 4-year-olds than adults showed explicit awareness of the underlying sequence structure, suggesting that such awareness is not sufficient to explain age-group differences in sequence learning.

Alternatively, recent research with adults with mild cognitive impairment suggests that multiple learning mechanisms may be engaged within a single short block of an implicit learning task (Nemeth et al., 2013). It is possible that children may show similar dissociations between early and later learning within individual SRT tasks blocks, although there is not specific evidence to suggest that utilization of early vs. late learning mechanisms would vary dramatically across fixed- vs. self-paced tasks. However, future studies should examine whether developmental differences in sequence-specific learning can be explained by isolated differences in particular learning mechanisms engaged earlier in a task block.

Developmental differences in the way children and adults approach the SRT task may also be responsible for observed group differences in implicit learning. For example, attempts by younger children to explicitly learn the underlying sequence structure may have a detrimental effect on implicit learning measures, due to overall reduced processing capacities in children vs. adults (Howard and Howard, 2001). Experimental support for this idea has been provided by Karatekin et al. (2007) who found that while children, adolescents, and adults showed equivalent learning on an SRT task under incidental learning conditions, when participants were explicitly instructed about the presence of a sequence, children then showed reduced learning in comparison to adults. In the present experiment we did not attempt to determine if age-related differences in strategies utilized during the SRT task impacted implicit learning measures.

It is also important to recognize that although we found robust differences in learning between children and adults, this result is not always replicated in the developmental implicit learning literature. In this study, we used z-normalized mean difference scores to assess sequence-specific learning, given the large group differences in average reaction time between children and adults. Overall there has been little consistency in how learning is quantified and compared for groups of individuals that differ in baseline reaction time. Various studies have compared raw reaction time measures (e.g., De Guise and Lassonde, 2001), raw reaction time differences between sequence and random trials (e.g., Meulemans et al., 1998; Fischer et al., 2007), proportional change in raw reaction time (e.g., Thomas and Nelson, 2001; Karatekin et al., 2007), as well as z-normalized difference scores (e.g., Thomas et al., 2004) across groups. At this point it remains unclear which method is most appropriate for correcting for group-based differences in baseline reaction time (e.g., see Janacsek et al., 2012 for a recent discussion of this topic). We contend that z-normalizing at the level of the individual subject is the most effective method of reducing the impact of age group differences in overall reaction time while preserving the rich distribution of reaction times for every participant. Importantly, the within age-group comparisons reported here, which are less affected by such transformations, indicate that modifications to the SRT paradigm that do not impact learning in adults can significantly affect implicit learning in younger children, even after accounting for baseline differences in reaction time.

### Task demand effects

Although previous studies have reported that implicit sequence learning is robust across procedural variations in adults (e.g., Meulemans et al., 1998; Robertson and Pascual-Leone, 2001; Chambaron et al., 2006; Deroost and Soetens, 2006; Song et al., 2007), our results demonstrated that preschool-aged children were highly sensitive to SRT task demands. Specifically, increased magnitude of sequence specific learning was observed in preschool-aged children for self-paced compared to fixed-paced trials. Future studies will be needed to determine at what age the effects of task pacing on learning become less salient. However, these overall results support findings in the more general implicit learning literature which suggest that alterations to the timing aspects of implicit or statistical learning task can impact the magnitude of learning observed (e.g., Frensch and Miner, 1994; Soetens et al., 2004; Toro et al., 2005; Turk-Browne et al., 2005; Arciuli and Simpson, 2009). Furthermore, related work examining the developmental course of associative learning in conditioning paradigms also argues that developmental differences in learning may be due to sensitivity to timing parameters. Specifically, although adult animals and humans showed similar rates of associative learning across conditioning paradigms, younger animals and human infants and children failed to show conditioning at equivalent rates under procedures in which a stimulus-free interval separated the conditioned and unconditioned stimulus or under long-delay conditions (see review in Herbert et al., 2003).

Although pacing and/or timing seems to be an important determinant of learning, future studies are also needed to isolate what characteristics of the self-paced learning environment (e.g., differences in attention, perceived agency, motivational demands, timing, stimulus-response contingencies, etc.) drive the developmental effects of task pacing on implicit learning. Other work has demonstrated that implicit learning can be separated into multiple components, including learning of stimulus-stimulus, response-response, and stimulus-response/response-stimulus contingencies (e.g., Ziessler, 1998; Ziessler and Nattkemper, 1998). Furthermore, when independently separated, certain types of contingencies, particularly learning the relationships between a response and a subsequent stimulus, may contribute more overall to learning than stimulus-stimulus or response-response associations. These separable types of learning cannot be independently evaluated in the present data set. In fact, the response-stimulus contingencies are comparable across all three tasks in our study when participants are making correct responses. However, one could argue that the response-stimulus contingencies are disrupted in both the fixed- and non-contingent self-paced tasks since the sequence of stimulus presentation is not affected by incorrect responses. If these disruptions affect learning, we would expect reduced learning in both the fixed-paced and non-contingent self-paced tasks. Instead, the maximal different in learning occurs between the fixed-paced and the self-paced tasks. Alternatively, the response-stimulus contingencies may also be affected by variations in task pacing. In the fixed-paced task there is a delay between the response and the subsequent stimulus because the task is non-contingent by design, where as in the self-paced task this is mostly absent. This potential difference in response effects could alter the learnability of the response-stimulus event pairs in the two task types, given that in the self-paced task events are linked more closely in contingency and time, which should support better learning (e.g., Frensch and Miner, 1994).

It is also possible that differences in attentional demands between the fixed-paced and self-paced paradigms are responsible for the difference in learning by task observed only in preschool-aged children. For example, Bulf et al. (2011) found that in a visual statistical learning paradigm, infants only showed evidence of detecting statistical regularities when the number of items in the learning sequence was reduced from six to four, suggesting that differences in selective attention and/or information processing strongly impact statistical learning in developmental populations. Although engagement of attention to other tasks can negatively impact implicit learning in adults (e.g., Toro et al., 2005), the effects of attention allocation during the SRT task have not been well studied developmentally. Our experiments did not utilize a dual-task or divided attention paradigm, but it is possible that the fixed- and self-paced tasks differed in global attentional or information processing demands for 4-year-old children while remaining relatively equivalent for adult participants.

While differences in task pacing did not impact adults' learning, for preschoolers the perception of agency and maintaining individual control over the pacing of a task may be intrinsically more motivating and/or rewarding than responding to fixed-paced stimuli. In other learning contexts, adults are known to prefer cues that predict opportunities to execute agency and make choices (vs. cues that do not convey a choice opportunity; Leotti and Delgado, 2011), even if the ability to make a choice conveys no additional reward (Bown et al., 2003). This preference likely arises because both perceiving and exercising control is highly adaptive for cognitive and emotional regulation throughout the lifespan (Leotti et al., 2010). In the present study we did not empirically assess whether children found the self-paced task versions more engaging than the controlled stimulus presentation rate of the fixed-paced task. However, it is notable that a high number of 4-year-old children were excluded from the fixed-paced condition due to poor accuracy or failure to complete the task, suggesting that maintaining attention and motivation to complete this task version was particularly difficult for children, perhaps because of a perceived lack of agency induced by the fixed-paced context.

To our knowledge no study has investigated the potential effects of motivation or perceived reward on implicit learning using an SRT-like task in children. In the adult literature, a recent study that rewarded participants monetarily for responding rapidly to target stimuli during an SRT paradigm found that extrinsic reward was associated with enhanced magnitude of sequence specific learning effects in comparison to punishment or control conditions (Wächter et al., 2009). Similarly, research with non-human primates has also indicated that altering reward schedules can impact implicit learning measures in the context of an SRT task (Procyk et al., 2000). Although we did not employ a reward schedule in our SRT task, experiment 2 in the present study provides preliminary evidence that the inclusion of accuracy feedback, and the possible changes induced in perceived reward for children, does not additionally benefit children's implicit learning within the self-paced learning context.

### Conclusion

In conclusion, the present study provides evidence for developmental differences in implicit sequence learning that are dependent on SRT task demands. It is likely that the large differences observed in implicit learning between fixed- and self-paced trials for preschool-aged children reflect broader differences in motivation, attention, reward, and/or perceived agency between the task rather than pure effects of stimulus timing. To better understand the potential role of task demands in developmental differences in sequence learning, it will be critical to determine what mechanisms are responsible for developmental improvements in implicit learning. fMRI studies comparing children and adults have suggested that there are both age- and learning-related neural correlates of SRT task performance (Thomas et al., 2004). Furthermore, brain regions such as the striatum and frontal-striatal circuits that support implicit learning in the context of the SRT task during adulthood (e.g., Rauch et al., 1997) are known to undergo extended development, well into later childhood (e.g., Sowell et al., 1999). Beyond implicit learning this diffuse network contributes to diverse functions such as attention allocation and motivated learning (e.g., Shohamy, 2011), which we hypothesize also contribute to observed differences in learning. Thus, we hope this study will drive researchers to consider at both behavioral and neurodevelopmental levels how task demands are related to developmental change in implicit learning.

## Author contributions

Kathleen M. Thomas, Julie C. Markant, and Amanda S. Hodel developed the research hypotheses and study design. Kathleen M. Thomas programmed the task paradigms. Jenie M. Cirilli-Raether, Sara E. Van Den Heuvel, and Amanda S. Hodel contributed to recruitment and data collection from child and adult participants. Julie C. Markant, Amanda S. Hodel, and Sara E. Van Den Heuvel implemented preliminary data processing and analysis procedures. Amanda S. Hodel completed final analyses and drafted the manuscript. All authors contributed to the editing and refinement of the manuscript.

## Conflict of interest statement

The authors declare that the research was conducted in the absence of any commercial or financial relationships that could be construed as a potential conflict of interest.
