# Supplementary material for: Developmental differences in effects of task pacing on implicit sequence learning
Source: Front Psychol. 2014 Feb 25;5:153. doi: 10.3389/fpsyg.2014.00153 (PMC3934418; doi:10.3389/fpsyg.2014.00153)

**Supplemental Figures 1-2**

Z-normalized reaction times for sequence and random trials by block for adults and preschoolers in the fixed- and self-paced conditions.


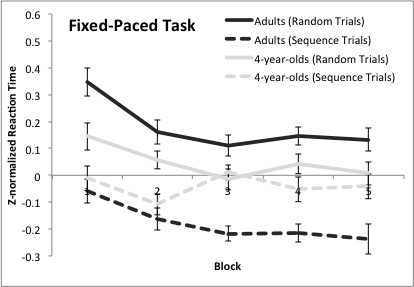


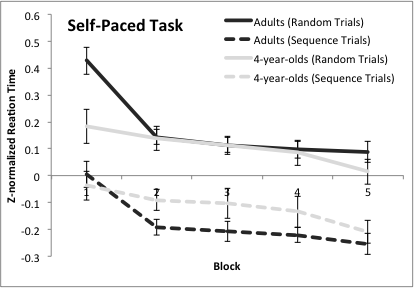


**Supplemental Figures 3-4**

Raw reaction times for sequence and random trials by block for adults and preschoolers in the fixed- and self-paced conditions.


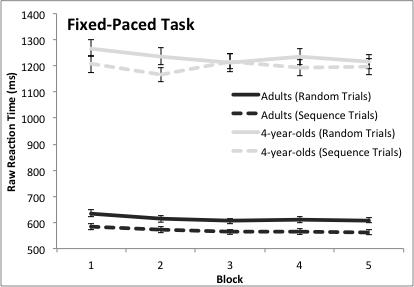


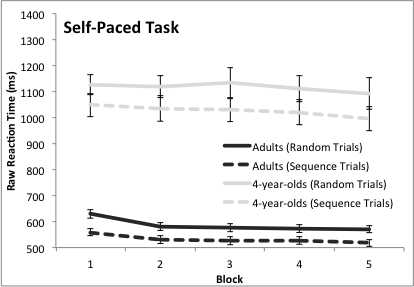


**Supplemental Figure 5**

Z-normalized reaction times for sequence and random trials by block for the non-contingent self-paced task.


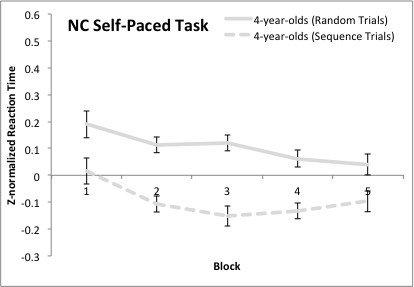


**Supplemental Figure 6**

Raw reaction times for sequence and random trials by block for the non-contingent self-paced task.


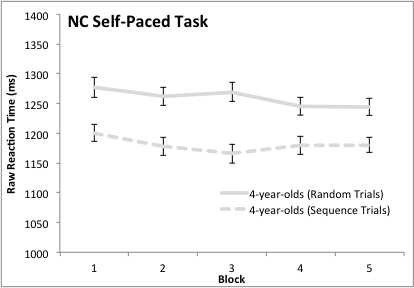

Supplement: Supplementary file 1 [file DataSheet1.DOCX]
